# Firmicutes Levels in the Mouth Reflect the Gut Condition With Respect to Obesity and Early Childhood Caries

**DOI:** 10.3389/fcimb.2021.593734

**Published:** 2021-05-27

**Authors:** Karina Ferreira Rizzardi, Claudia Maria dos Santos Pereira Indiani, Renata de Oliveira Mattos-Graner, Emerson Tavares de Sousa, Marinês Nobre-dos-Santos, Thaís Manzano Parisotto

**Affiliations:** ^1^ Laboratory of Clinical and Molecular Microbiology, University São Francisco, Bragança Paulista, Brazil; ^2^ Department of Oral Diagnosis, Piracicaba Dental School, State University of Campinas, Piracicaba, Brazil; ^3^ Department of Health Sciences and Pediatric Dentistry, Piracicaba Dental School, State University of Campinas, Piracicaba, Brazil

**Keywords:** dental caries, obesity, child, *Firmicutes*, *Bacteroidetes*, feces, dental plaque

## Abstract

The present cross-sectional study investigated whether Firmicutes (F) and Bacteroidetes (B) levels in the mouth reflected the gut condition in obesity and early childhood caries (ECC). Eighty preschoolers (3-5 years) were equally assigned into four groups: 1. obese + ECC, 2. obese + caries-free (CF), 3. eutrophic + ECC, and 4. eutrophic + CF. Nutritional status and ECC were assessed based on the WHO criteria. Dental biofilm and fecal samples were collected for F and B quantification using RT-PCR analysis. Data were evaluated using three-way-ANOVA and Pearson’s correlation (α = 0.05). Regardless of the anatomical location effect (*p* = 0.22), there were higher values for F in the obese children + ECC compared with those in obese + caries-free (CF) in both mouth and gut (*p* < 0.05). The correlation for F at these sites was negative in obese children + ECC (r = −0.48; *p* = 0.03) and positive in obese children + CF (r=0.50; *p* = 0.03). Bacteroidetes were influenced by ECC (*p* = 0.03) and the anatomical location (*p* = 0.00), and the levels tended to be higher in the mouth of the obese children + ECC (*p* = 0.04). The F/B ratio was higher in the gut and was affected by the anatomical location (*p* = 0.00). This preliminary study suggested that modulated by ECC, counts of oral Firmicutes reflected corresponding condition in the gut of obese preschoolers. In addition, we first evidenced that the Firmicutes phylum behave differently according to the nutritional status and caries experience and that supragingival biofilm and gut could share levels of similarity.

## Introduction

Diverse microorganisms are capable of colonizing different body parts, particularly the gut (Mills et al., 2019) and mouth, where they are highly abundant. The oral cavity is the gateway to the body. During the mastication process, various types of food are ground by teeth and mixed with saliva before reaching the stomach and intestine (Dewhirst et al., 2010). Approximately 10^11^ bacterial cells are ingested daily with saliva (Socransky and Haffajee, 2000). Thus, bacteria present in the mouth may contribute to the flora of the lower digestive tract; however, the mechanism by which the oral bacteria survive the defense barriers of the gastrointestinal tract remains unclear. Interestingly, even if these bacteria do not survive, dead bacteria might stimulate several pathogens in the gut and create a new phenotype by upregulating the microbial virulence genes (Olsen and Yamazaki, 2019).

Changes occurring in the gut microbiota, such as an increase in Firmicutes and a decrease in Bacteroidetes, have been reported to play a pivotal role in the pathogenesis of obesity (Gallardo-Becerra et al., 2020). Gut microorganisms can protect the intestinal mucosa and regulate the fermentation and absorption of polysaccharides obtained through the daily diet, which are closely linked to fat accumulation (Muscogiuri et al., 2019). Importantly, when compared with caries-free (CF) children, children with early childhood caries (ECC) harbor different bacteria in their oral cavity, such as high amounts of *Streptococcus mutans, Steptococcus sobrinus, Veillonella parvula*, and *Scardovia* spp. (a newly described pathogen) (Dashper et al., 2019). Lactobacilli are more prevalent among caries-active children versus CF ones (Parisotto et al., 2015). Notably, *Lactobacillus* spp. and *Streptococcus mutans* belong to the Firmicutes phylum. Recent studies have suggested that Firmicutes shifts to higher numbers in obese children (Riva et al., 2017; Gallardo-Becerra et al., 2020).

Obesity and caries are highly prevalent and have serious consequences on children’s life and health. A caries active child could have intense pain, infection, chewing difficulties, loss of school days, and poor oral health-related quality of life. In contrast, obese children are prone to develop serious comorbidities and diseases related to obesity, such as cardiovascular diseases, cancer, and diabetes, which are responsible for using a substantial part of the National Health Expenditure every year (de Oliveira et al., 2015).

Therefore, studies investigating obesity and ECC, together with the dysbiosis associated with these diseases, are promising as they can identify the common risk indicators. Moreover, a possible connection between the oral and gut microbiota in obese children has not been reported. More importantly, childhood is a crucial period characterized by significant changes, providing unique opportunities for microbial intervention targeting health promotion (Hollister et al., 2015; Riva et al., 2017). Thus, new information in this regard is extremely valuable and a recent systematic review published by our group pointed out that changes in Firmicutes and Bacteroidetes phyla are remarked indicators for childhood obesity (Indiani et al., 2018). The present investigation aimed to assess whether Firmicutes and Bacteroidetes levels in the mouth were capable of reflecting the gut condition with respect to obesity and ECC.

## Material and Methods

### Ethical Considerations and Sample Characteristics

This observational study was approved by the Ethical Committee in Research of the São Francisco University (protocol number: CAAE-42997115.4.0000.5514) in full accordance with the World Medical Association Declaration of Helsinki. Parents or guardians provided informed consent for their children’s inclusion. As part of a larger study involving 968 children, 80 children (3–5 years and 11 months) were assigned to the following groups after clinical examination for dental caries and obesity diagnosis: 1) obese + ECC (*n* = 20), 2) obese + CF (*n* = 20), 3) eutrophic + ECC (*n* = 20), and 4) eutrophic + CF (*n* = 20).

The two-sided equality of variances hypothesis (H0: µ1 − µ2 = 0 versus H1: µ1 − µ2 ≠ 0; H0: µ1 = µ2 versus H1: µ1 ≠ µ2) and sample recruitment was used to calculate the number of volunteers according to the following formula (Chow et al., 2003):

$$N=\frac{{\left(Z_{\frac{\propto }{2}}+Z_{\beta }\right)}^{2}\theta^{2}}{{\left(\mu 1-\mu 2\right)}^{2}}$$

where Z critical for α was 0.05 (type I error), Z critical for β was 0.20 (type II error), μ1 − μ2 indicates the mean difference between the mean ratio of gut levels of Firmicutes and Bacteroidetes in eutrophic (1.06) and obese (0.48) children, and θ represents the expected variation value (0.38) of the eutrophic group (due to their higher variability). Data from the study of Xu et al. (2012) were compatible with the study proposal and, consequently, with the sample forecast. Therefore, approximately 18 volunteers per group were required. This number was increased (+0.1) to 20, considering the sample losses during data collection and analysis.

Considering the characteristics of the experimental design and statistical modeling based on two independent factors (disease and nutritional status), a subgroup was created to screen the possible significant differences according to the caries experience. From a pragmatic perspective, the sample size was equally factorial distributed as eutrophic children and obese children (ECC and CF children, each with “*n*” equal to 20). Thus, the sample size of 40 children per group was used for both eutrophic and obese children according to their nutritional status.

The inclusion criteria were healthy preschoolers of both genders, eutrophic or obese, and who were able to cooperate with the clinical examinations. Children who were absent from preschool during the dental examinations/anthropometric measures, who were classified as underweight, overweight, or at risk of being overweight, with systemic diseases under steroid medicines treatment, and who were taking antibiotics or probiotics during the sample collection period were excluded from this study.

All children enrolled in this study attended public preschools in the urban and fluoridated areas of Bragança Paulista, SP, Brazil. Moreover, they had similar access to fluoridated dentifrice and were of similar socioeconomic status. They spent most of the day at preschool, where the content of all meals was standardized.

### Anthropometric Measures

For weight and height measurements, a calibrated electronic scale, a non-extensible measuring tape affixed to a wooden board at a 90° angle to the ground, and a movable piece as a headboard, were used. For the height measurements, children were placed in an upright position, with the Camper Plane parallel to the floor, and with their feet slightly apart. Preschoolers were weighed standing erect, on marked footprints in the center of the scale, and arms stretched to the sides of the body. Even though the children were weighed wearing only extremely light uniforms and were barefoot, the uniform weight was subtracted from the final measure.

The body mass index (BMI = weight [kg]/height [m^2^]) was used to classify the children’s nutritional status (eutrophic or obese) according to the World Health Organization guidelines (World Health Organization and Multicentre Growth Reference Study Group, 2006; World Health Organization, 2018). Children aged 5 years or younger were considered obese when they had >+3 Z-score and eutrophic if they had ≥−2 Z-score and ≤+1 Z-score. Children aged 5 years, 1 month up to 5 years, 11 months were classified as obese when they had ≥+2 Z-score and eutrophic if they had ≥−2 Z-score and ≤+1 Z-score.

All measurements were performed using two previously calibrated examiners (Kappa interexaminer = 0.82).

### Collection of Dental Biofilm

The presence of clinically visible dental biofilm was identified as a white/yellowish biomass close to the gingival margin. Dental biofilm was collected from all dental smooth surfaces after at least 1 h of food intake. The collection was performed at preschools, with a dentin spoon excavator. The collected biofilm was placed in preweighed microtubes and transported into refrigerated boxes to the Laboratory of Clinical and Molecular Microbiology of the University São Francisco, where they were frozen at −80°C until DNA extraction and microbiological analysis, using real-time polymerase chain reaction (qRT-PCR).

### Clinical Examination

The teeth were cleaned and dried with gauze, and dental caries was diagnosed under head-set light, with a clinical mirror and a ball-ended dental probe, according to the WHO criteria (decayed, missing, or filled surfaces) (World Health Organization, 2013), modified by the inclusion of early carious lesions (Parisotto et al., 2010). The clinical examinations were performed by two dentists, using a previously calibrated gold standard examiner, who received all the theoretical and practical instructions regarding the criteria to be used. During the calibration process, approximately 10% of the sample was reexamined at 1-week intervals. To guarantee reliability in interexaminer assessment, the Kappa statistics were calculated with a value of 0.86, indicating an excellent agreement between the examiners.

### Stool Sample Collection

Children received labeled universal plastic containers for carrying out stool sample collection in their own homes. Written instructions were given to their guardians to ensure that the stool did not touch the inside of the toilet. Samples were kept refrigerated in hermetically sealed plastic bags placed inside home refrigerators, for no more than 12 h, until they were taken to the preschool. From home to preschool (divided by regions) it took about 5–10 min, as the children enrolled lived nearby. The preschool stool specimens were immediately placed into refrigerated boxes and were transported within 2h to the Laboratory of Clinical and Molecular Microbiology of the University São Francisco, where they were frozen at −80°C until further analyses.

### DNA Extraction of Biofilm and Stool Samples

For the DNA extraction process, 5 mg of biofilm/stool samples were suspended in 1 mL of 0.9% NaCl and were then subjected to centrifugation (11,000 g/10 min). About 300 µL of Tris-EDTA (50 mM/10 mM of biofilm/stool samples) was added to the bacterial pellet for dissolution. Subsequently, 50 µL of 20% sodium dodecyl sulfate (SDS) was added and samples were heated at 70°C for 8 min. Thereafter, the supernatant was discarded and about 0.12 g of 0.1 mm diameter silica/zirconia beads (BioSpec Products, Bartlesville, OK, USA) were added to the pellet to promote the mechanical breakdown of bacterial cells in Mini-Beadbeater equipment (BioSpec Products, Bartlesville, OK, USA) at maximum power for 1 min and at room temperature. Next, 100 µL of 5 M NaCl and 80 µL of 10% cetyltrimethylammonium bromide (CTAB) were added to the samples, followed by 750 µL of a mixture of phenol:chloroform: isoamyl alcohol (25:24:1, pH 8.0). Then, the samples were centrifuged at 11,000 g/5 min. The supernatant was transferred to a new microtube. This step was repeated once. Next, a mixture of chloroform: isoamyl alcohol (24:1, pH 8.0) was added and the samples were centrifuged at 11,000 g/5 min. Furthermore, 4 µL of RNAse A was added to the supernatant and incubated at 40°C for 15 min. After further shaking, the cells were incubated at 40°C for 25 min. Thereafter, 75 µL of 3 M potassium acetate and 800 µL of isopropanol were added, followed by inverse mixing (6–10×) and stored at −20°C for 20 min. Then, the microtubes were centrifuged at 11,000 g/10 min, and the supernatant was removed, leaving the DNA pellet drying, with the microtubes facing downward, at room temperature (40 min). Eventually, 40 µL of TE buffer (pH 8.0) was added to the obtained DNA, and samples were frozen at −20°C (Stipp et al., 2013). The DNA concentration was analyzed using spectrophotometry and was adjusted to a final concentration of 50 ng/µL (Biodrop µLite Spectrophotometer, Biochrom US Inc., Holliston, MA, USA). The integrity of DNA samples was examined using electrophoresis analysis using 1% agarose gel, stained by the Unisafe dye (20,000× – Uniscience, Osasco, SP, Brazil).

### Real-Time Quantitative Polymerase Chain Reaction (qRT-PCR)

qRT-PCR was performed using the 7300 Real-Time System (Applied Biosystems, Foster City, CA, USA) with SYBR Green Power up (Thermo Fisher Scientific, Carlsbad, CA, USA) using plates for 96 samples. Primer sequences have already been described in the scientific literature (forward primer Firmicutes, 5′-GGAGYATGTGGTTTAATTCGAAGCA-3′ and reverse primer, 5′-AGCTGACGACAACCATGCAC-3′; forward primer Bacteroidetes, 5′-GGARCATGTGGTTTAATTCGATGAT-3′ and reverse primer 5′-AGCTGACGACAACCATGCAG-3′) and were used to generate a 126-bp amplicon (Guo et al., 2008). It is important to emphasize that primers for Firmicutes and Bacteroidetes were selected based on conservative 16S rRNA gene sequences of a multiplicity of gut/bucal species (GenBank- http://www.ncbi.nlm.nih.gov/genbank/) of Firmicutes and Bacteroidetes phyla, respectively. All primers were evaluated for their specificity in the software BLAST (Basic Local Alignment Search Tool - http://www.ncbi.nlm.nih.gov/blast/), and have confirmed to be specific for their purposes.

For each qRT-PCR reaction, 10 μL of the reaction mixture, including 5 µL of SYBR Green Power up (Thermo Fisher Scientific, Carlsbad, CA, USA), 2.9 µL of H_2_O, 1.5 µL of DNA sample, and 0.3 µL of each primer were used. Amplification and detection were performed according to the following cycles: 2 min at 50°C, 10 min at 95°C, 40 cycles of 15 s at 95°C, and 1 min at 60°C (Guo et al., 2008).

*Clostridium perfringens* (ATCC 13124) and *Bacteroides fragilis* (ATCC 25285) were used as positive controls for Firmicutes and Bacteroidetes phyla, respectively. DNA samples of control strains were serially diluted by ten-fold (five dilution points; 10^1^–10^5^ ng/μL) to generate standard curves for determining the absolute target quantity in samples. More specifically, the software (Sequence Detection Software version 1.3.1, Applied Biosystems, Foster City, CA, USA) measures the amplification of the target in both the standard dilution series and test samples. A standard curve was generated using data from the standard dilution series. According to the standard curve, the software interpolates the absolute quantity of the target in the test samples.

The critical threshold cycle had the detectable fluorescence above the background (standard threshold: 0.200). All qRT-PCR for the standards and DNA samples were performed in duplicate.

### Statistical Analysis

Data normality and homogeneity of variances were tested using the Shapiro–Wilk test and Levine test, respectively. Variables that violated the premises of analysis of variances were transformed (Samal et al., 1999); thus, data from Bacteroidetes were transformed by square root and that from the Firmicutes/Bacteroidetes ratio (F/B) were reciprocally transformed.

The main interaction effects of the three factors (1. anatomical location: oral cavity or gut; 2. nutritional status: obese or eutrophic; and 3. disease: caries or CF) among the dependent variables (Firmicutes, Bacteroidetes, and F/B) were investigated using the three-way factorial analysis of variance model. Levine’s test was used to prove the equality of multiple variance-covariance matrices considering the level of significance of 0.05. Moreover, alpha (α), beta (β), and partial eta squared (ηp²) represent, respectively, the type I error, the type II error, and the strength of each effect. Pairwise comparisons were performed between children with ECC and CF children within each condition (1. obese + ECC; 2. obese + CF; 3. eutrophic + ECC and eutrophic + CF) after Bonferroni adjustment for multiple comparisons.

Pearson correlation analysis was performed to determine the strength and direction of the relationship between Firmicutes and Bacteroidetes in the mouth and gut.

Data were statistically analyzed using the SPSS package for Windows, version 21.0 (SPSS, Inc., Chicago, IL, USA).

## Results

**Table 1** illustrates the three-way ANOVA design listing the main effects of nutritional status (eutrophic × obese), disease (CF × ECC), and anatomical location (mouth x gut) of the Firmicutes, Bacteroidetes, and Firmicutes/Bacteroidetes ratio in children of all groups: 1. obese + ECC; 2. obese + CF; 3. eutrophic + ECC; and 4. eutrophic + CF. The interaction effects were also displayed.

**Table 1:** Interaction effect of nutritional status (eutrophic × obese) and disease (CF × ECC) on the Firmicutes and Bacteroidetes levels (ng/µL) in the mouth and gut.

Anatomical Location	Nutritional Status	Disease	Firmicutes		Bacteroidetes		F/B	
			Mean (SD)		Mean (SD)		Mean (SD)	
Mouth	*Eutrophic*	*CF*	*2578.13 (1215.8)a*		*4903.05 (2740.3)a*		*0.52 (0.15)a*	
		*ECC*	*2205.06 (944.0)a*		*4355.72 (1641.9)a*		*0.50 (0.14)a*	
	*Obese*	*CF*	***1849.05 (1242.9)a***		***3936.29 (2538.2)a***		*0.46 (0.13)a*	
		*ECC*	***2693.83 (1317.8)b***		***5516.69 (2550.8)b***		*0.48 (0.14)a*	
*Gut*			***Mean (SD)***		***Median (IQR)***		***Median (IQR)***	
	*Eutrophic*	*CF*	*2086.55 (1238.3)a*		*577.00 (1036.2)a*		*3.20 (9.61)a*	
		*ECC*	*2586.33 (1300.7)a*		*815.50 (1681.9)a*		*3.17 (6.04)a*	
	*Obese*	*CF*	***1551.55 (940.2)a***		*345.04 (1482.9)a*		*2.88 (6.59)a*	
		*ECC*	***2206.31 (999.6)b***		*633.58 (2010.9)a*		*3.02 (8.02)a*	
*Three-way Factorial ANOVA design*			***α (β)***	**ηp²**	***α (β)***	**ηp²**	***α (β)***	**ηp²**
*Main Effects*	*Anatomical Location*		*0.22 (0.24)*	*0.010*	***0.00 (1.00)***	***0.564***	***0.00 (1.00)***	***0.490***
	*Nutritional Status*		*0.12 (0.34)*	*0.016*	*0.73 (0.06)*	*0.001*	*0.60 (0.08)*	*0.002*
	*Disease*		***0.03 (0.59)***	***0.031***	***0.03 (0.57)***	***0.030***	*0.94 (0.05)*	*0.000*
*Interaction Effects*	Anatomical Location*Disease		*0.37 (0.15)*	*0.005*	*0.67 (0.07)*	*0.001*	*0.46 (0.12)*	*0.000*
	Anatomical Location*Nutritional Status		*0.37 (0.14)*	*0.005*	*0.94 (0.05)*	*0.000*	*0.72 (0.06)*	*0.001*
	Disease*Nutritional Status		*0.06 (0.47)*	*0.023*	*0.29 (0.18)*	*0.007*	*0.96 (0.05)*	*0.000*
	Anatomical Location*Nutritional Status*Disease		*0.16 (0.29)*	*0.013*	0.11 (0.36)	*0.017*	*0.53 (0.09)*	*0.003*
*Levine’s Test*			***0.684***		***0.252***		***0.248***	

Statistical analyses were performed with a sample of 80 volunteers. ηp², Partial eta squared; F/B, Ratio of Firmicutes to Bacteroidetes. Data with Gaussian distribution are expressed as mean (SD) and that with non-Gaussian distribution are expressed in median (IQR, interquartile range). Bacteroidetes data were transformed by square root, and data of F/B ratio were reciprocally transformed. The bold type indicates statistical significance. Different small letters represent significant pairwise differences between early childhood caries (ECC) and caries-free (CF) children after Bonferroni adjustment for multiple comparisons. Graphic representations of analysis of variance interactions are provided in **Supplementary Material**, **Graphic S1** (Firmicutes), **Graphic S2** (Bacteroidetes), and **Graphic S3** (F/B).

Regarding Firmicutes levels, a statistically significant effect of the disease (p = 0.03) was not influenced by anatomical location and by nutrition status alone in the combined three-factor matrices. However, the interaction effect between disease and nutritional status demonstrated an α value at the margin of statistical nonsignificance (p = 0.06), which could be interpreted with caution since the effect size (type II error) and the strength (partial eta squared) of this significance are weak (the profile plots did not run in parallel while considering the nutritional status versus caries status in the mouth, **Graphic S1C**). Pairwise comparisons demonstrated a statistically significant difference (represented by the distinct letters in the table, p < 0,05), between CF and ECC in the group of obese children, with higher amounts under the ECC condition, at both anatomical locations (mouth and gut), as evidenced by **Graphic S1B**.

For Bacteroidetes levels, the main effect evidenced a significant influence of the anatomical location (p = 0.00) and the disease (p = 0.03) on the expression of Bacteroidetes, which tended to be higher in the mouth of obese children with ECC (significant pairwise comparison: p = 0.043) compared with obese caries-free children (**Graphic S2B**).

The F/B ratio was only influenced by the anatomical location (p = 0.00), which was higher in the gut than that in the mouth (**Graphic S3B**). In addition, the ratios were very similar regarding ECC *versus* CF in obese or eutrophic children, in both mouth and gut, and no significant differences were observed in pairwise comparisons (represented by the same letters in the table, p > 0,05).

A strong and positive correlation was observed between Firmicutes and Bacteroidetes in the oral cavity of all groups (r = 0.73 to 0.83). Moreover, the relationship between Firmicutes in the mouth and the gut could be observed only in the group of obese children, which expressed positive and negative correlations in CF (r = 0.50) and ECC (r = -0.48) children, respectively. There was a negative correlation between Firmicutes in the gut and Bacteroidetes in the mouth (**Table 2**).

**Table 2:** Pearson’s correlation models for obese and eutrophic children according to their ECC status.

Eutrophic		Obese	
CF	ECC	CF	ECC
F_M_ x B_M_= 0.84 (p<0.0001)	F_M_ x B_M_= 0.73 **(p<0.0001)**	F_M_ x B_M_= 0.76 **(p<0.0001**)	F_M_ x B_M_= 0.83 **(p<0.0001)**
		F_M_ x F_G_= 0.50 **(p=0.026)**	F_M_ x F_G_= -0.48 **(p=0.034)**
			B_M_ x F_G_ = -0.48 **(p=0.033)**

CF, caries-free; ECC, early childhood caries; FM, Firmicutes mouth levels; FG, Firmicutes gut levels; BM, Bacteroidetes mouth levels. Considering the best fit for normal distribution and variance homogeneity, the data of Bacteroidetes were transformed by taking the square root. The bold type indicates statistical significance.

## Discussion

Obesity and ECC are diseases with important implications for health systems worldwide (Di Cesare et al., 2019). It was estimated that children with high BMI scores had 2.15 times more chances of experiencing ECC than eutrophic children (Manohar et al., 2020). Both are considered as non-communicable diseases that share a common risk factor (unhealthy eating behavior and high carbohydrate intake); however, from a holistic perspective, the relationship between these conditions may not be simple. For caries, strong evidence suggests a log-linear dose-response relationship between fermentable carbohydrate intake and dental caries (Sheiham and James, 2015); whereas for obesity, the synergic influence of sugar consumption and fat-rich nutrients may be more meaningful (World Health Organization, 2018; Liberali et al., 2020).

Moreover, looking beyond the causality hypothesis between obesity and ECC, one of the most intriguing possibilities suggests the influence of the microbiome. To date, dysbiosis of the gut environment (in obese children) and disruption of homeostasis in supragingival biofilms (in ECC) could sustain this point of view. To address this question, we used a cohort of children that share almost 70% of the same diet (preschool-based meals program) to investigate whether the levels of two of the most abundant phyla in the gut and oral cavity (Firmicutes and Bacteroidetes) behave differently according to the nutritional status and caries experience. Furthermore, considering the anatomical connection between the oral cavity and gut, we tested the hypothesis that these two distinct niches (supragingival biofilm and gut) could share levels of similarity.

It was demonstrated for the first time that Firmicutes levels in the mouth seem to reflect the gut condition only in obese preschoolers (**Tables 1**, **2** and **Graphic S1B**). Specifically, a positive correlation was observed between Firmicutes levels in the mouth and gut in CF children, whereas a negative correlation was observed in ECC affected children. This difference in the phylum profile could provide insights for understanding the microbial variation throughout the digestive tube and for exploiting the phenomena of coexistence between caries and obesity. In both anatomical locations (mouth and gut), significantly higher levels of Firmicutes were found in obese ECC children *versus* obese CF children. The nutritional status (obesity condition) presumably favored the difference between the disease status (CF × ECC), leading to a notable statistical difference (with moderate power and effect strength). This finding is reinforced by the α value close to statistical significance regarding the interaction effect between disease and nutritional status (**Table 1**).

Remarkably, it is important to recognize that the shift from a healthy microbiota to a more pathogenic one is influenced by the host eating habits in a process called ecological adaptation and that the majority of Firmicutes species are involved in fermentation pathways. A recent study investigating connections between the oral microbiome and severe ECC reported that Firmicutes was the major phylum found in caries active dentinal microbiota (Hurley et al., 2019). In a similar trend, Firmicutes in the gut has been reported to increase in childhood obesity (Da Silva et al., 2020; Gallardo-Becerra et al., 2020). Such a scenario is difficult to distinguish based on our sample stratification and dual-niche-based disease-level variance matrix.

Firmicutes is a huge bacterial phylum and comprises over 250 genera including *Lactobacillus, Mycoplasma, Bacillus, Clostridium, and Streptococcus* (Bajzer and Seeley, 2006). Lactobacilli and mutans streptococci, for example, have the metabolic ability to produce large amounts of acid and are highly acidogenic, which is eventually linked to dental tissue demineralization and dental caries initiation/development (Parisotto et al., 2010; Ma et al., 2015); moreover, these bacteria can survive at extremely low pH. Furthermore, the metabolism of some intestinal microorganisms belonging to the Firmicutes phylum may facilitate the extraction and storage of calories from the diet (Bajzer and Seeley, 2006; Muscogiuri et al., 2019), enabling the hydrolysis of indigestible polysaccharides to glucose, that is, activating lipase by the direct action of the villous epithelium (Riva et al., 2017). Intriguingly, our results demonstrate that the caries status switches the direction of the correlation between Firmicutes in the mouth and the gut of obese children from a positive (for CF children) to a negative one (for ECC children). Notably, the use of dental biofilm samples from upper anterior smooth surfaces as a nonshedding highly informative niche of caries disease and predominance of Firmicutes could underscore this inverse relationship. In this respect, it may be hypothesized that caries lesions provide significant retentive niches for bacteria accumulation, diminishing the availability of bacteria to be swallowed and throughout the digestive path reach the intestine.

A significant and strong influence was found between the anatomical location and caries disease in Bacteroidetes (**Table 1**, **Graphic S2B**), and this finding can provide a unique insight into the role of Bacteroidetes in the chaotic transition of oral to gut microbiota. For instance, Bacteroidetes levels were higher in the mouth of obese ECC children (significant pairwise comparison: p = 0.043) than those in obese CF children. Bacteroidetes was found to be the second most prevalent phylum in the oral microbiome of children suffering from severe ECC in both dentinal and salivary samples (Hurley et al., 2019). Moreover, some species belonging to Bacteroidetes, such as *Prevotella* spp., have been reported to be associated with ECC (Wang et al., 2019). More specifically, *Prevotella* spp. are gram-negative rod-shaped anaerobes with potential proteolytic/amino acid degrading activities, which play a pivotal role in caries development when the dentin tissue is exposed and the matrix is partly degraded (Takahashi and Nyvad, 2016).

In the gut, although our data did not demonstrate a significant p-value for Bacteroidetes pairwise comparisons (perhaps due to the lower power to detect differences after familywise error corrections), 84% higher expression of this phylum was found when the obese ECC children were compared with obese CF children. This is a splendid reason to believe that the high data dispersion hides possible data significance. Nonetheless, other relevant information such as dental biofilm acidogenicity, virulence factors (glycosyltransferases), and metabolic profile could confound in contributing to each phenotype explored in this study. Furthermore, the interaction of Bacteroidetes with Firmicutes could be another source of variability.

Even though differences between the F/B ratio regarding the ecological niches (gut and mouth) are remarkable, the results of the present investigation did not reveal a particular representative influence of nutritional status and ECC over this ratio. Similarly, the early investigation of Chen et al. (2020), indicated no tendency regarding the proportions of F/B phyla in childhood obesity. In contrast, the influence of the F/B ratio in obesity has been reported in previous studies (Riva et al., 2017; Indiani et al., 2018). Moreover, in the intestinal microbiota of obese children, the F/B ratio has been reported to increase (Bervoets et al., 2013), and conversely to decrease (Liang et al., 2020). Conflicting results could be linked to the population characteristics, that is, geographical origin, age, feeding habits, and sample molecular management methodology.

Oral bacteria have been permanently established in the oral environment since the third day after birth and are modified after tooth eruption (Caufield et al., 2000). Microorganisms play a crucial role in the digestion process, which starts in the mouth and ends in the gut. Noticeably, our data demonstrated a strong and positive correlation between Firmicutes and Bacteroidetes in the oral cavity in all groups. More importantly, this result implies that both phyla might cooperate even within a fermentable dysbiotic niche. Li and Ma (2020) argued that F and B ally against Actinobacteria (represented by several opportunistic pathogens). In the gut microbiota, the functional variations between F and B seem to be more complex.

Physiological aspects of health and disease can be better understood and new opportunities can be created to translate data into clinical practice within the scope of a clinical study. However, the present investigation had certain limitations. First, since this was a cross-sectional study, no causality could be inferred. Second, the number of participants was restricted. In this respect, we argue that three-way ANOVA has a complex statistical structure (with a robust and extremely conservative rank test of interaction) and no previous data was available with a similar source of variation (factors) to assess the expected variability and appropriate calculation of a powerful sample size. Thus, this is a pioneering study investigating the oral and gut microbiota collectively in obese children with ECC, and our preliminary data should be used to design larger studies. Considering that the prevalence of obesity in the young child population is low (about 6%–8%), a larger geographic area or multiple cities should be encouraged.

Outstandingly, by 2030, obesity is expected to affect over a billion people (Kelly et al., 2008) and ECC already affects 12%–90% of worldwide children (WHO, 2016). Both diseases are preventable *via* appropriate management of common risk factors, and healthy habits can be established at the preschool age, thereby providing special opportunities for intervention (Hollister et al., 2015; Riva et al., 2017).

Furthermore, the fact that oral samples may reflect fecal samples concerning a determined bacterial phylum in young children is quite interesting because dental biofilm collection is easy and can be performed at any time. In contrast, stool samples are usually collected at home, relying on parents/guardians’ compliance.

This preliminary study suggested that modulated by ECC, counts of oral Firmicutes reflected corresponding condition in the gut of obese preschoolers. In addition, we first evidenced that the Firmicutes phylum behave differently according to the nutritional status and caries experience and that supragingival biofilm and gut could share levels of similarity.

## Data Availability Statement

The raw data supporting the conclusions of this article will be made available by the authors, without undue reservation.

## Ethics Statement

The studies involving human participants were reviewed and approved by Ethical Committee in Research of the São Francisco University . Written informed consent to participate in this study was provided by the participants' legal guardian/next of kin.

## Author Contributions

Conceptualization: TP, RM. Methodology: TP. Validation: KR. Formal analysis: ES. Investigation: KR, CI. Resources: TP. Data curation: KR. Writing-original draft preparation: KR and TP. Writing-review and editing: MN-d-S, ES, TP. Visualization: ES. Supervision: TP. Project administration: TP. Funding acquisition: TP. All authors contributed to the article and approved the submitted version.

## Funding

This study was supported by FAPESP (2015/24600-2) and CNPq (409475/2016-5) grants.

## Conflict of Interest

The authors declare that the research was conducted in the absence of any commercial or financial relationships that could be construed as a potential conflict of interest.
