# Supplementary material for: Firmicutes Levels in the Mouth Reflect the Gut Condition With Respect to Obesity and Early Childhood Caries
Source: Front Cell Infect Microbiol. 2021 May 27;11:593734. doi: 10.3389/fcimb.2021.593734 (PMC8190403; doi:10.3389/fcimb.2021.593734)
Supplement: Supplementary file 1 [file DataSheet_1.docx]

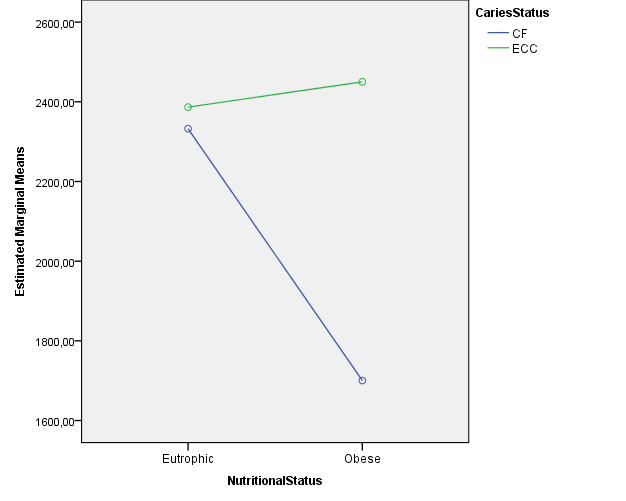

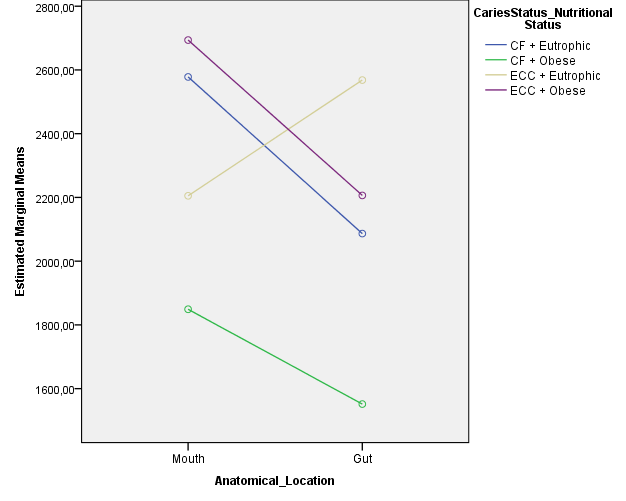


**B**

**A**


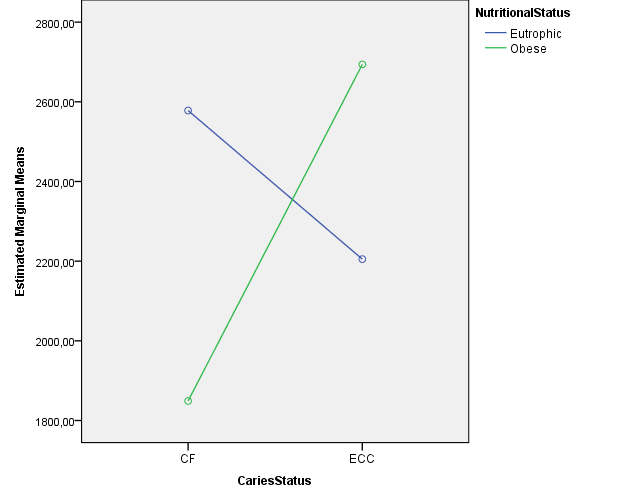

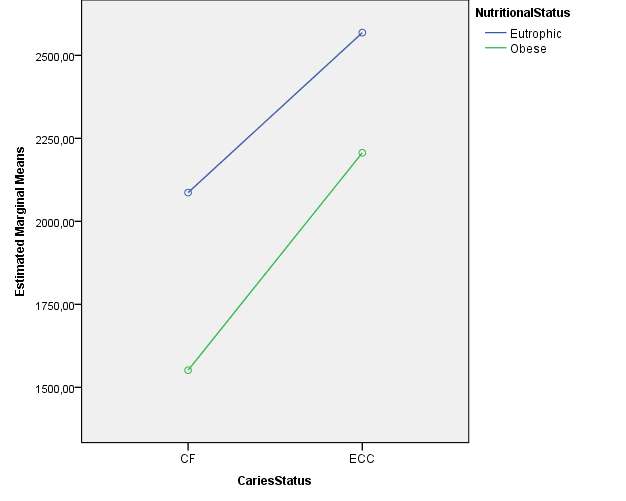


**C**

**D**

**Graphic S1:** Interaction between nutritional status and disease (A), disease plus nutritional status and anatomical location (B), disease and nutritional status for mouth (C), and disease and nutritional status for gut (D) on the Firmicutes levels.


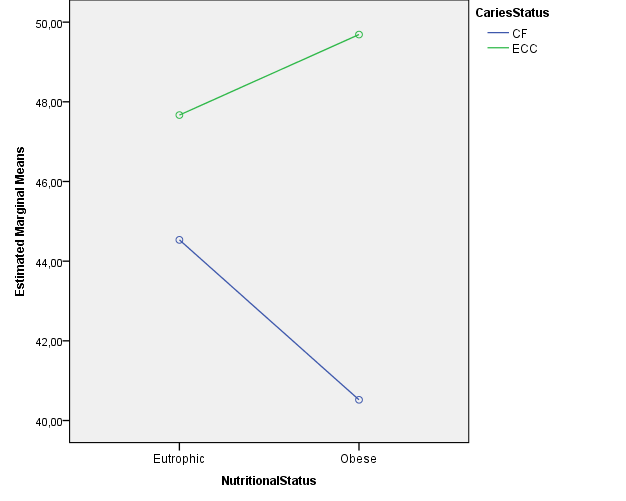

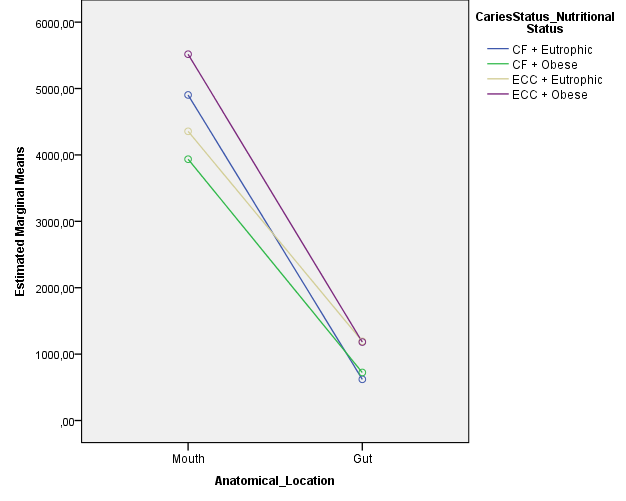


**B**

**A**


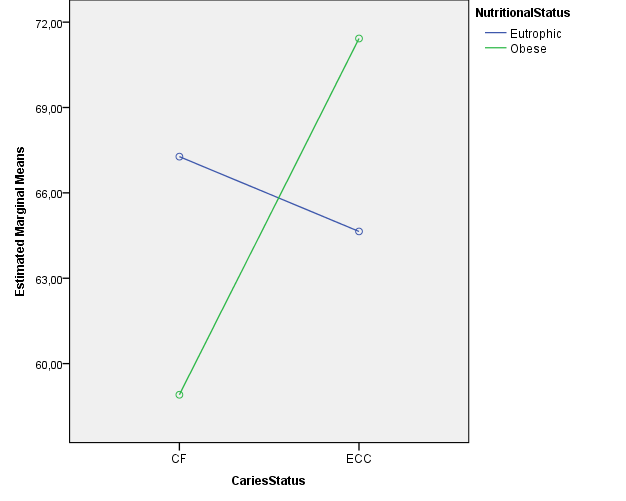

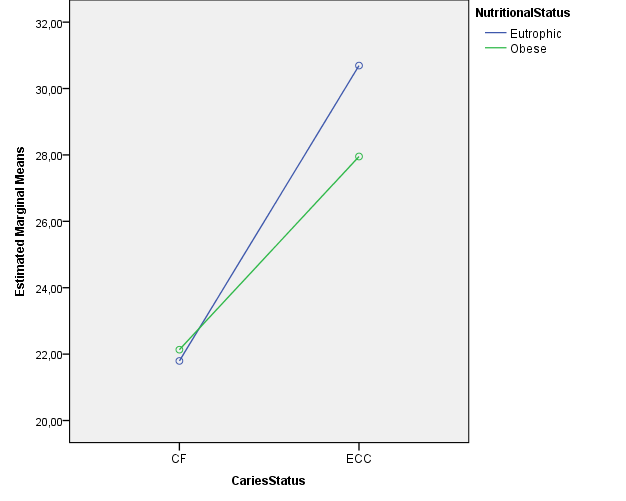


**D**

**C**

**Graphic S2:** Interaction between nutritional status and disease (A), disease plus nutritional status and anatomical location (B), disease and nutritional status for mouth (C), and disease and nutritional status for gut (D) on the Bacteroidetes levels.


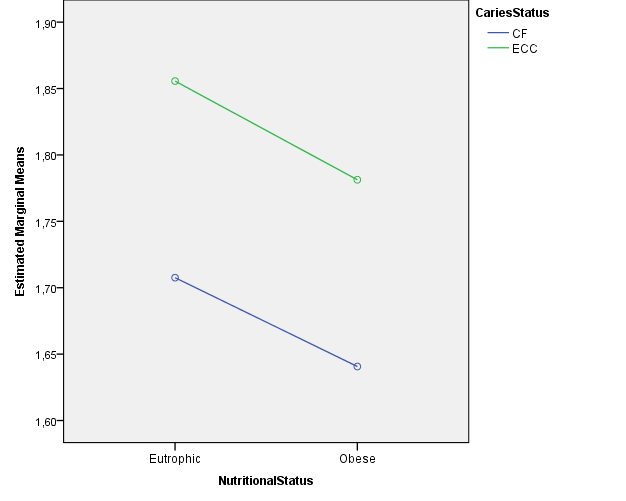

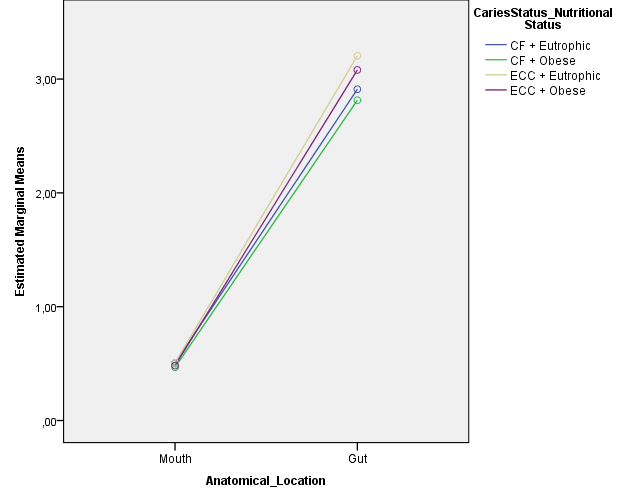


**A**

**B**

**C**

**D**


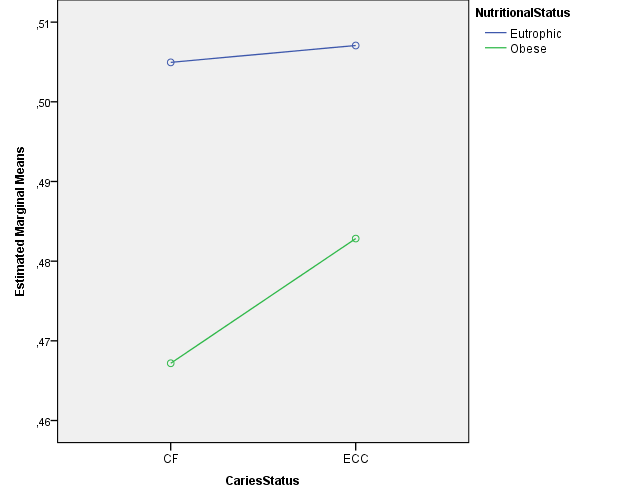

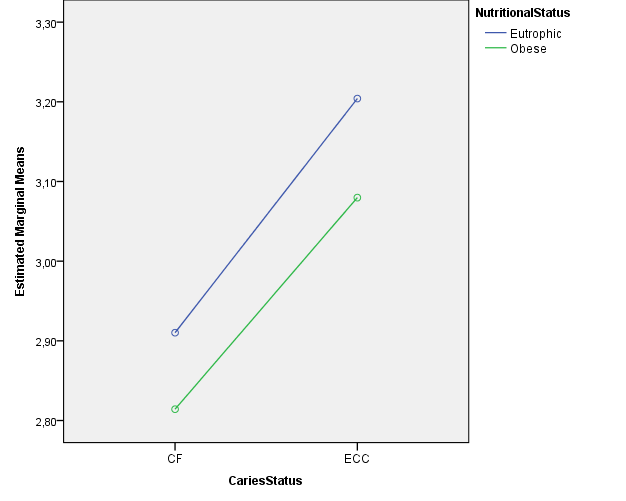


**Graphic S3:** Interaction between nutritional status and disease (A), disease plus nutritional status and anatomical location (B), disease and nutritional status for mouth (C), and disease and nutritional status for gut (D) on the Firmicutes/Bacteroidetes ratio.
